# Towards an ontology of cognitive processes and their neural substrates: A structural equation modeling approach

**DOI:** 10.1371/journal.pone.0228167

**Published:** 2020-02-10

**Authors:** Teal Eich, David Parker, Yunglin Gazes, Qolamreza Razlighi, Christian Habeck, Yaakov Stern

**Affiliations:** 1 Leonard Davis School of Gerontology, University of Southern California, Los Agneles, California, United States of America; 2 Cognitive Neuroscience Division, Department of Neurology, Columbia University, New York, New York, United States of America; University of Pittsburgh, UNITED STATES

## Abstract

A key challenge in the field of cognitive neuroscience is to identify discriminable cognitive functions, and then map these functions to brain activity. In the current study, we set out to explore the relationships between performance arising from different cognitive tasks thought to tap different domains of cognition, and then to test whether these distinct latent cognitive abilities also are subserved by corresponding “latent” brain substrates. To this end, we tested a large sample of adults under the age of 40 on twelve cognitive tasks as they underwent fMRI scanning. Exploratory factor analysis revealed 4-factor model, dissociating tasks into processes corresponding to episodic memory retrieval, reasoning, speed of processing and vocabulary. An analysis of the topographic covariance patterns of the BOLD-response acquired during each task similarity also converged on four neural networks that corresponded to the 4 latent factors. These results suggest that distinct ontologies of cognition are subserved by corresponding distinct neural networks.

## Introduction

Two longstanding challenges in the field of cognitive neuroscience are to (1) isolate cognitive functions, and (2) map brain activity to these cognitive functions [1]. Since the advent of functional brain imaging, tens of thousands of fMRI studies have been conducted, attempting to investigate the neural pathways that lead to specific behavioral outcomes. To synthesis and aggregate the results from individual studies, different forms of “informatics-driven” approaches have been developed [1–4]. One of the most commonly used approach is the meta-analysis, in which data from many studies that each use different tasks to probe a particular cognitive function—working memory training [5] or social rejection [6], for example—are pooled to look for common behavioral patterns of results or neural substrates. While meta analyses provide important topographic neural localization of cognitive abilities, the approach is inherently top-down: it is assumed that tasks that purportedly tap the cognitive process of interest really do tap that process, and therefore that brain areas that are commonly activated across these different tasks inferentially index that process.

Recently, several groups have used different data-driven approaches to uncover the neural networks underlying different cognitive abilities. Yeo and colleagues [7], for example, leveraged data from the BrainMap database [8], which includes over 10,000 imaging experiments, to create a “latent cognitive structure and its topography” ([7], p. 3665–6). They explored functional specialization and flexibility across 83 different cognitive tasks and reported meaningful functional specialization across tasks (e.g., tasks which in the extant literature are commonly thought to tap inhibitory processes, including the Simon, Posner, Stroop, Flanker, N-Back and Task Switching, all recruited a common neural network), but also reported that individual networks can demonstrate remarkable flexibility (e.g., specific cortical regions participated in multiple cognitive components and divergent cognitive tasks). Bertolero and colleagues [9–11] extended this work in several important ways using graph theory network based approaches, first showing with resting state functional MRI data that distinct modules perform distinct cognitive functions, but that, as the number of cognitive functions within a given experimental task increases, so too does activity in connector nodes that link modules [9]. Bertolero and colleagues further showed that hub connectivity accurately predicts performance across 4 different cognitive tasks, and found that those individual who had hubs with higher participation coefficients (e.g., hubs with greater diversity in modular connections) as opposed to higher hub strength (hubs with stronger modular connection) showed better cognitive performance [10,11]. Our group has also published several reports that attempt to flesh out the neural networks associated with performance in a data driven manner that is free from assumptions about cognitive categories. For example, we empirically tested whether four purported domains of cognition—based on the results of studies of several thousand adults across the adult lifespan who were administered extensive batteries of cognitive tasks to both separate into different categories of cognition, and account for performance differences on numerous other cognitive tasks [60, 61, 62]—would show unique spatial covariance patterns of brain activity [16]. Linear indicator regression was used to derive covariance patterns from data collected while older and younger participants completed 12 different cognitive tasks thought to index episodic memory retrieval, reasoning, speed of processing and vocabulary. In this analysis, while the derivation of covariances patterns for each of the four cognitive domains was “forced” to be optimal across age groups, the pattern expressions were found to predict the appropriate cognitive domain of each task out of sample. Habeck and colleagues [12] recently extended this covariance approach to also include behavioral performance, reporting covarying brain areas that not only predicted the appropriate cognitive domain with high probability, but also strongly correlated with behavioral performance.

Here, we were interested in exploring whether, from a bottom up perspective, the data, without extensive supervision or covariance pattern analysis, reveal unique patterns of brain activations that correspond to each cognitive domain. To this end, we used exploratory factor analysis (EFA)—a type of multivariate and data-driven structural equation modelling [13] that can statistically determine the underlying major sources of variance and thus the structure of a set of variables [14]—to test for behavioral ontologies of cognitive function, and the neural networks that are related to these ontologies [15].

We used GLM analyses to capture task-related activation for as participant performed 12 cognitive tasks, and measured performance on each of the tasks. We first determined the best fitting model characterizing the latent structure of the behavioral performance arising from these 12 different tasks. We then tested the best fitting model that characterized the “latent” structure of task related BOLD-activation. To this end, we used an approach similar to that used with the behavioral data, except we tested whether the correlations between the magnitude of neural activation within brain areas that topographically overlapped across each combination of task pairs showed a similar latent structure: a set of areas that showed the same inter-correlated task-related activations within a given cognitive domain, along with a covariance pattern that was not expressed (or minimally expressed) during the performance of tasks associated with the other cognitive domains. For both the behavioral and neuroimaging data, this approach allowed us to not only test for convergent validity—whether specific sets of tasks indeed relate to each other or share common variance, but also to test for discriminant validity: whether tasks that are related to each other are also simultaneously *less* related to the tasks outside of their category. Finally, we assessed brain-behavior relationships across these two latent constructs. This approach, unlike the ones we have previously used, avoids issues of over-fitting since task-design is not used in any manner to derive unique patterns of activation. Further, the analysis is done at the voxel level, facilitating the localization of function similar to that used in standard atlases of the voxel activation associated with particular tasks.

## Methods

### Participants

Analyses included data from one hundred and fourteen healthy volunteers (72 female and 37 male) ranging in age from 19–40 (average age = 30.08 (SD = 5.35), average years of education = 15.94 (SD = 2.37; range = 9–24 years) who had participated in a large, ongoing study in the Department of Neurology at Columbia University, the Reference Ability Neural Network study [16]. All participants were required to be native English speakers, right-handed, and have at least a fourth-grade reading level. Participants were recruited via random-market-mailing, and screened for MRI contraindications and hearing or visual impairment that would impede testing. The Columbia University Medical Center Human Subjects IRB specifically approved this study under Protocol AAAI2752. Written Informed Consent for all participants was obtained prior to testing, and participants received compensation for participation in the study.

### Procedure

Imaging data were acquired on 3.0 Tesla Philips Achieva Magnet. Each participant underwent fMRI scanning while performing 12 computerized tasks, described in detail below. Tasks were administered over the course of two 2-hour scanning sessions on different days, with six tasks administered in each scanning-session. One session presented three tasks thought to index episodic memory retrieval and three tasks thought to index reasoning, also interspersed in a fixed order: logical memory, paper folding, word order recognition, matrix reasoning, paired associates, and letter sets. The other session presented three tasks thought to index vocabulary and three tasks thought to index speed, interspersed in a fixed order: synonyms, digit-symbol, antonyms, letter comparison, picture naming, and pattern comparison. Seventy-nine percent of participants completed both sessions (72 participants completed all 12 tasks, 10 participants completed 11 tasks, 4 participants completed 10 tasks, 3 participants completed 8 tasks, and 1 participant completed 5 tasks). Nine participants completed the memory/reasoning session and the first task from the vocabulary/speed session, while 1 completed all tasks from the vocabulary/speed session and only the first task from the memory/reasoning session. Three additional participants only completed the memory/reasoning session, while 8 only completed the vocabulary/speed session. One participant completed the first 3 memory/reasoning tasks, and 1 vocabulary task, one participant completed all vocabulary/speed tasks expect the picture naming task, and 2 participants only completed the picture naming task.

The order of tasks within session was not varied, but the order of the two sessions was counterbalanced across participants. Prior to each scan session, computerized training was administered for the six tasks to be administered during that session. At the completion of training for each task, participants had the option of repeating the training. During training, responses were on the computer keyboard. During scans, they were made on the LUMItouch response system (Photon Control Company). For all tasks except for picture naming, task responses are made on a LUMItouch response system and behavioral response data were recorded on the task computer. The picture naming test utilized a verbal response recorded from an in-scanner microphone, from which behavioral performance was determined after the scan.

Task administration and data collection were controlled by a computer running EPrime software, and electronically synchronized with the MR scanner. Task stimuli were back-projected onto a screen located at the foot of the MRI bed using an LCD projector. Participants viewed the screen via a mirror system located in the head coil, and, if needed, had vision corrected to normal using MR compatible glasses (manufactured by SafeVision, LLC. Webster Groves, MO). Task onset was electronically synchronized with the MRI acquisition computer.

#### Cognitive tasks

The following 12 cognitive tasks were administered to participants in the scanner.

#### Synonyms [17]

Participants completed 15 trials in which a capitalized probe word was presented at the top of the screen, with four numbered choices below. Participants were instructed to match the probe word to its synonym or to the word most similar in meaning as quickly and accurately as possible. The total task was 6 min and 26 s long, with 3 items in each of 5 blocks for a total of 15 items. Each block lasted 42 s, with each item presented for 13.5 s and a 500 ms interstimulus interval (ISI) between items. A 36 s fixation cross was presented at the start of the task, and a 28 s fixation cross was interspersed between blocks.

#### Antonyms [17]

This task was identical to the Synonyms task, except participants were instructed to match the probe word to its antonym, or to the word most different in meaning.

#### Picture naming

In this task, participants were presented with 40 colored bitmap images, adapted from the picture naming task of the WJ-R Psycho-Educational battery [18, 19]. Participants were instructed to verbally name the pictures. Audio recordings of responses were filtered using a custom adaptive noise filtering procedure, and then transcribed and scored. The entire scan was 6 min and 16 s long, consisting of five 40 s blocks, with 8 stimuli in each block. Each stimulus was presented for 4.5 s with a 500 ms ISI. A 36 s fixation cross was presented at the start of the task, and a 28 s fixation cross was interspersed between blocks.

#### Digit symbol

A code table was presented on the top of the screen, consisting of the numbers 1–9, each paired with an associated symbol. Below the code table an individual number/symbol pair probe was presented. Participants were instructed to indicate whether each of 90 individual pairs was the same as that in the code table using a differential button press. Participants were instructed to respond as quickly and accurately as possible. The entire scan was 7 min and 4 s long, consisting of 5 blocks, with 18 items in each block. Each item was presented for 2.5 s with an ISI of 250 ms. A 36 s fixation cross was presented at the start of the task, then a 28 s fixation was interspersed between blocks.

#### Letter comparison [20]

Participants were instructed to indicate whether two 3–5 letter strings, presented alongside one another, were the same or different. There were 60 total trials. The task, which contained five 42 s blocks, lasted a total of 6 min and 26 s. Each block consisted of 12 items, each presented for 3 s with an ISI of 500 ms. A 36 s fixation cross was presented at the start of the task, then a 28 s fixation was interspersed between blocks.

#### Pattern comparison [20]

This task was identical to the Letter Comparison task, except participants were instructed to indicate whether two figures, consisting of varying numbers of lines connecting at different angles, presented alongside one another, were the same or different.

#### Paper folding [21]

Participants were shown a pictorial representation a piece of paper being folded, in which a hole was punched through in the last image of the folded paper in the sequence. Participants had to decide which of 5 options represented the pattern of holes if the paper was unfolded. The task was 14 min and 26 s long and began with a 24 s fixation-cross, followed by the first stimulus, which stayed on screen for between 11 and 85 s. If a response was made in the first 11 s, the stimulus terminated. If a response was made after 11 s, the stimulus was terminated immediately after the response. If no response was made, the stimulus terminated at 85 s. The minimum number of trials presented was 7 and the maximum was 18, depending on each participant’s response times. The ISI was 35 s.

#### Matrix reasoning [22]

Participants were given a matrix of abstract figures, divided into nine cells, in which the figure in the bottom right cell was missing. Below the matrix, they were given eight figure choices, and were instructed to choose which of the figures would best complete the missing cell. The task timing was identical to that of the Paper Folding task.

#### Letter sets [21]

Participants were presented with five sets of letters, where four out of the five sets had a common rule (e.g. no vowels), with one of the sets not following this rule. Participants were instructed to select the unique set. The task timing was identical to that of the Paper Folding task.

#### Logical memory

This task required participants to remember specific details from stories presented on the computer screen. The participant was asked to answer detailed multiple-choice questions about the story, with four possible answer choices. The task was 7 min long, and consisted of 2 stories, with ten questions per story. Each story was divided into three 1 to 2 sentence sections, with each section displayed for 10 s. Ten seconds after completion of the story, the questions were presented for 10 s with a 2.5 s ISI between questions. Thirty-second fixation-crosses were presented before and between the stories.

#### Word order recognition

Participants were instructed to remember the order of twelve words, each presented one at a time on the screen for 4 s. A variable ISI, between 700 ms and 11.4 s, occurred between each word. Participants were then given a probe word at the top of the screen, and four additional word choices below. Each probe was presented for 6 s followed by a 2 s ISI. Participants were instructed to select the word that had *immediately* followed the probe word in the list. The task had two word lists, with ten questions following each list. The total task duration was 7 min 2 s with 30 s fixation at the beginning of task and between the two word lists.

#### Paired associates

In this task, six pairs of words were presented on the screen, one pair at a time, for 2 s with a variable ISI of 200 ms to 5.6 s. Participants were instructed to remember the pairs. Following the pairs, they were given a probe word at the top of the screen and four additional word choices below. The probe and choices are presented for 5 s with an ISI of 2 s. Participants were asked to choose the word that was originally paired with the probe word. The task contained two lists of pairs, with six probe questions in each list. The task contains two lists of pairs, with six probe questions for each list. The task lasted a total of 3 min and 24 s, with 30 s fixation at the beginning of the task and 10s fixation between the two lists.

### Behavioral factor structure analysis

Exploratory factor analysis (EFA) with Geomin (oblique) rotation was performed on the behavioral data using MPlus software [23]. The indicators consisted of each participant’s behavioral performance data for each task, described above. For all tasks except digit symbol, letter comparison and pattern comparison, the proportion of correct responses, excluding trials where the participant did not respond (i.e. timed out), were used. For digit symbol, letter comparison and pattern comparison, the reaction time (RT) on correct trials was used. Factor loadings were assessed according to guidelines set by [24] who suggested that loadings below 0.32 should be considered poor, 0.45 fair, 0.55 good, 0.63 very good and loadings above 0.71 excellent. To compare the structure and statistical fit parameters we interrogated the Comparative Fit Index (CFI; [25]), Tucker-Lewis Index (TLI; [26]); the Standardized Root Mean Square Residual (SRMR); the Root Mean Square Error of Approximation (RMSEA) [27]; and the Chi-Square test for the three, four and five factor models.

### Image analysis procedures

#### Image acquisition

At each session, a scout, T1-weighted image was acquired to determine participant position. Participants underwent a T1-weighted MPRAGE scan to determine brain structure, with a TE/TR of 3/6.5 ms and Flip Angle of 8 degrees, in-plane matrix size of 256 x 256, field of view of 256 mm x 256 mm, and 180 slices in the axial direction with a slice-thickness/gap of 1/0 mm. For the EPI acquisition, the parameters were: TE/TR (ms) 20/2000; Flip Angle 72°; In-plane matrix size = 112x112; Field of view = 224 mm x 224 mm; Slice thickness/gap (mm) = 3/0; Slices = 41. In addition, MPRAGE, FLAIR, DTI, ASL, and a 7-minute resting BOLD scan were acquired. A neuroradiologist reviewed each participant’s scans.

#### Pre-processing

FMRIB Software Library v5.0 (FSL) and custom-written Python code was used to preprocess the imaging data. Each participant’s 12 task-activation fMRI scans were pre-processed in FSL [28] using the following steps: (1) within-subject histogram computation for each participant volume to identify noise (FEAT); (2) realignment of the fMRI scans was performed by rigid-body spatial registration of all the volumes to the middle volume (MCFLIRT); (3) slice-timing correction was performed by shifting the time-series for each slice to the instance when the middle slice was acquired; (4) brain-mask creation from first volume in subject’s fMRI data; (5) high-pass filtering (T = 128 sec) was performed with a non-linear Gaussian kernel with cut-off frequency of 0.01 Hz; (6) pre-whitening; (7) General-Linear-Model (GLM) estimation with equally temporally filtered regressors and double-gamma hemodynamic response functions; and (8) non-linear registration of functional and structural images with subsequent normalization into MNI space (FNIRT).

#### Subject-level time-series modeling

General linear models for each participant consisted of block-based time-series for the speed and vocabulary tasks, and event-related models for the reasoning and memory tasks. For the memory tasks, while both the encoding, retention and retrieval phases were imaged, only the retrieval phase was analyzed. A single regressor was used to compare task performance to an intrinsic baseline, defined one of two ways depending on the analysis. For block design task models, a boxcar model for each task block was used. The regressor was obtained by convolving this box car train with the canonical hemodynamic response function (HRF). The intrinsic baseline consisted of the interval between task blocks during which there no stimuli were present on the screen. For event related task models, the intrinsic baseline was modelled as the combination of all non-task periods. Each stimulus presentation was modeled from the onset of the stimulus to the response, using correct trials only, with the regressor obtained by convolving the stimulus presentation with the canonical HRF. For each participant’s 12 tasks, a standard GLM was run on each scan using the appropriate regressor to generate a parameter estimate (beta) map.

#### Group-level modeling

Group-level activation maps were created for each task by passing all standard-space subject-level beta values and variance into a standard FSL higher level analysis [29]. Cluster-wise analysis was carried out using FSL’s Gaussian random field theory based cluster analysis, identifying contiguous clusters with a z threshold of 2.3 clusters, corresponding to a probability *p*<0.05 after cluster-wise multiple comparisons correction. To assess the spatial similarity of activated regions between tasks, we calculated the spatial correlations of group-level z-statistics for every combination of task-pair. The spatial correlation of two z-statistic maps indexes the similarity of activation patterns within a region, across the task-pairs. To compare two tasks, we calculated the spatial correlation of the group level z-statistics from each task. Doing this for every combination of all 12 tasks resulting in a correlation matrix consisting of all 78 correlation values, which shows us the similarity of how two tasks activate the same region. We limited this calculation to areas with significant positive activations in both tasks (e.g., the intersection of positive activation masks from both tasks). The results from an analysis of the negative BOLD-response are the focus of a related manuscript that is currently in preparation.

#### Neuroimaging data factor structure analysis

In order to assess the factor structure of the neural data, EFA was performed on the correlation matrix the 78 z-statistics that resulted from the comparison of all 12 tasks’ significantly overlapping positive activations calculated in the Group level analysis. As with the behavioral factor analysis, model structure, loadings and statistical fit parameters we interrogated the CFI, TLI, SRMR, RMSEA and the Chi-Square test for the three, four and five factor models.

## Results

### Behavioral factor structure

**Fig 1:**
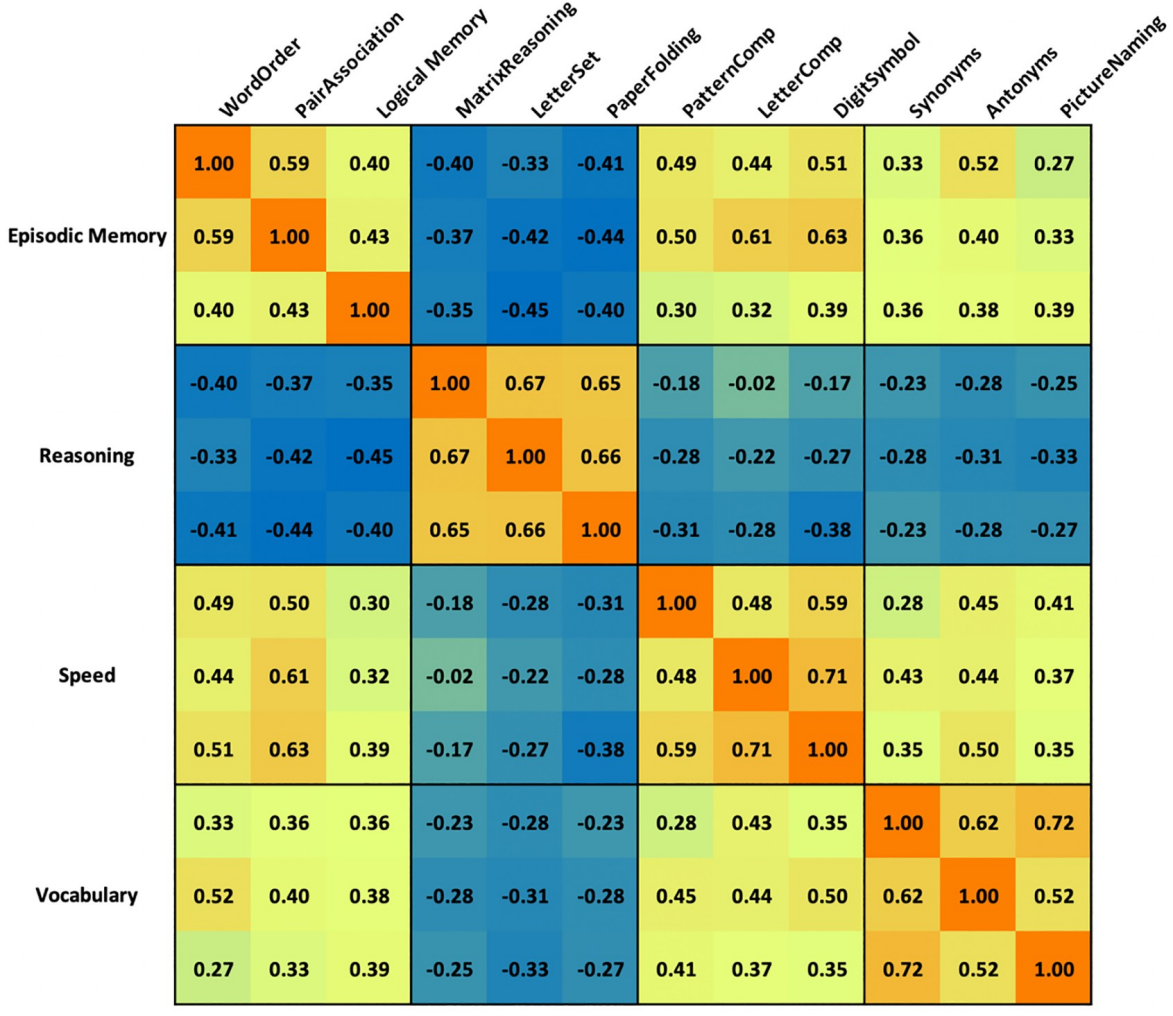
Color-coded correlation matrix obtained from behavioral data from the 12 cognitive tasks completed in the scanner. Warm colors indicate positive correlations. Cool colors indicate negative correlations.

The full correlation matrix of the behavioral data is shown in Fig 1. As can be seen in Table 1 (top), in all 3 models, the pattern comparison, letter comparison and digit symbol tasks factored onto a single latent variable (speed), and the synonyms, antonyms and picture naming tasks loaded onto a vocabulary factor. The three-factor model combined the memory retrieval and reasoning factors into one single factor, whereas the five factor model split the reasoning factor into two factors: spatial reasoning (consisting of the paper folding task) and non-spatial reasoning (consisting of the letter set and matrix reasoning tasks).

**Table 1:** Factor loadings from an exploratory factor analysis for a 3-, 4- and 5-factor model of (Top) the behavioral performance measures from the 12 cognitive tasks; and (bottom) the correlations between z-statistics of positively activated areas that topographically overlapped across task-pairs calculated from the group-level maps. Bolded values indicate factor-membership. Color shaded areas indicate cognitive domain membership by task based on previous literature: memory retrieval, reasoning speed and vocabulary.

Data	# Factors	Factor	Word Order	Pair Assoc	Mem	Mat Reas	Letter Set	Paper Fold	Patt Comp	Letter Comp	Digit Symb	Syn	Anton	Pic Name
Behavior	3	1	**0.564**	**0.695**	**0.376**	**0.855**	**0.824**	**0.643**	-0.025	-0.208	-0.283	0.387	0.495	0.38
		2	-0.578	-0.619	-0.533	-0.468	-0.341	-0.415	**0.821**	**0.78**	**0.782**	-0.384	-0.465	-0.405
		3	0.39	0.415	0.413	0.431	0.489	0.368	-0.242	-0.318	-0.258	**0.952**	**0.661**	**0.754**
	4	1	**0.884**	**0.628**	**0.416**	0.558	0.45	0.524	-0.434	-0.344	-0.408	0.356	0.566	0.27
		2	0.521	0.701	0.389	**0.844**	**0.852**	**0.617**	-0.068	-0.27	-0.346	0.398	0.471	0.396
		3	-0.48	-0.551	-0.505	-0.386	-0.274	-0.335	**0.826**	**0.815**	**0.805**	-0.333	-0.382	-0.38
		4	0.362	0.395	0.419	0.427	0.48	0.379	-0.248	-0.332	-0.257	**0.903**	**0.677**	**0.798**
	5	1	**0.854**	**0.67**	**0.43**	0.626	0.512	0.593	-0.409	-0.342	-0.412	0.373	0.595	0.297
		2	-0.476	-0.544	-0.5	-0.369	-0.265	-0.325	**0.83**	**0.815**	**0.805**	-0.332	-0.372	-0.369
		3	0.464	0.662	0.344	**0.788**	**0.904**	0.534	-0.012	-0.228	-0.307	0.387	0.419	0.346
		4	0.326	0.349	0.395	0.358	0.42	0.32	-0.257	-0.322	-0.242	**0.929**	**0.648**	**0.794**
		5	0.074	0.233	0.161	0.414	0.315	**0.668**	0.028	-0.177	-0.161	0.129	0.213	0.404
Imaging	3	1	**0.933**	**0.896**	**0.794**	0.476	0.507	0.377	0.58	0.548	0.625	0.707	0.737	0.433
		2	0.376	0.335	0.33	**0.888**	**0.871**	**0.939**	0.292	0.352	0.438	0.353	0.317	0.102
		3	0.649	0.6	0.529	0.555	0.442	0.36	**0.869**	**0.816**	**0.912**	**0.713**	**0.701**	**0.637**
	4	1	**0.913**	**0.936**	**0.779**	0.448	0.462	0.354	0.568	0.491	0.591	0.621	0.659	0.435
		2	0.399	0.355	0.347	**0.888**	**0.88**	**0.936**	0.288	0.351	0.437	0.361	0.325	0.101
		3	0.584	0.554	0.466	0.53	0.375	0.331	**0.899**	**0.778**	**0.892**	0.588	0.57	0.661
		4	0.661	0.575	0.586	0.424	0.488	0.313	0.538	0.658	0.633	**0.939**	**0.966**	**0.368**
	5	1	**0.916**	**0.934**	**0.789**	0.46	0.475	0.367	0.585	0.515	0.61	0.646	0.681	0.437
		2	0.413	0.371	0.353	**0.901**	**0.877**	**0.934**	0.313	0.359	0.461	0.363	0.326	0.13
		3	0.613	0.571	0.503	0.511	0.386	0.313	**0.895**	**0.829**	**0.89**	0.676	0.667	0.659
		4	0.667	0.583	0.588	0.409	0.476	0.295	0.531	0.649	0.621	**0.931**	**0.975**	0.368
		5	0.174	0.195	0.006	-0.004	-0.141	-0.183	0.217	-0.095	0.21	-0.03	-0.009	**0.593**

As can be seen in Table 2 (top), the fit statistics were better for the 4- factor models compared to the 3-factor model. While the 5-factor model had marginally better RMSEA and SRMSR values compared to the 4-factor model [30,31], the four-factor model both converges with previous findings [12,16,20,32–34] and is a more parsimonious model.

**Table 2:** Structural equation model fit statistics for a 3-, 4- and 5-factor model of (Top) the behavioral performance measures from the 12 cognitive tasks; and (bottom) the correlations between z-statistics of areas that topographically overlapped across task-pairs calculated from the group-level maps (bottom).

Data	# Factors	# Parameters	Chi-square			CFI	TLI	RMSEA	SRMR	Neg. Residual Variences
			X^2^	DF	P value					
Behavior	3	57	38.452	33	0.2363	0.99	0.98	0.038	0.03	no
	4	66	20.878	24	0.6459	1	1.016	0	0.021	no
	5	74	10.163	16	0.858	1	1.044	0	0.014	no
Imaging	3	45	14390	33	0.00	0.871	0.742	0.209	0.046	no
	4	54	2150	24	0.00	0.981	0.947	0.094	0.014	no
	5	62	1169	16	0.00	0.990	.0957	0.085	0.007	no

#### Neural factor structure

An exploratory factor analysis with goemin (oblique) rotation including a 3-, 4- and 5-factor model of the correlations between z-statistics of task-pairs calculated from the group-level maps (thresholded at z = 2.3) was then conducted. As can be seen in Table 1 (bottom), the 3-factor model produced a memory retrieval latent variable, a reasoning variable and a variable combining the parameter estimates for both the speed and vocabulary tasks. The 4-factor model produced a memory retrieval network, a speed network, a reasoning network and a vocabulary network. Factor loadings for each task except the picture naming task in the vocabulary latent network were high (>.778), with most >.9 [24]. The 5-factor model was the same as the 4-factor model, with the 5^th^ factor being picture naming alone.

**Fig 2:**
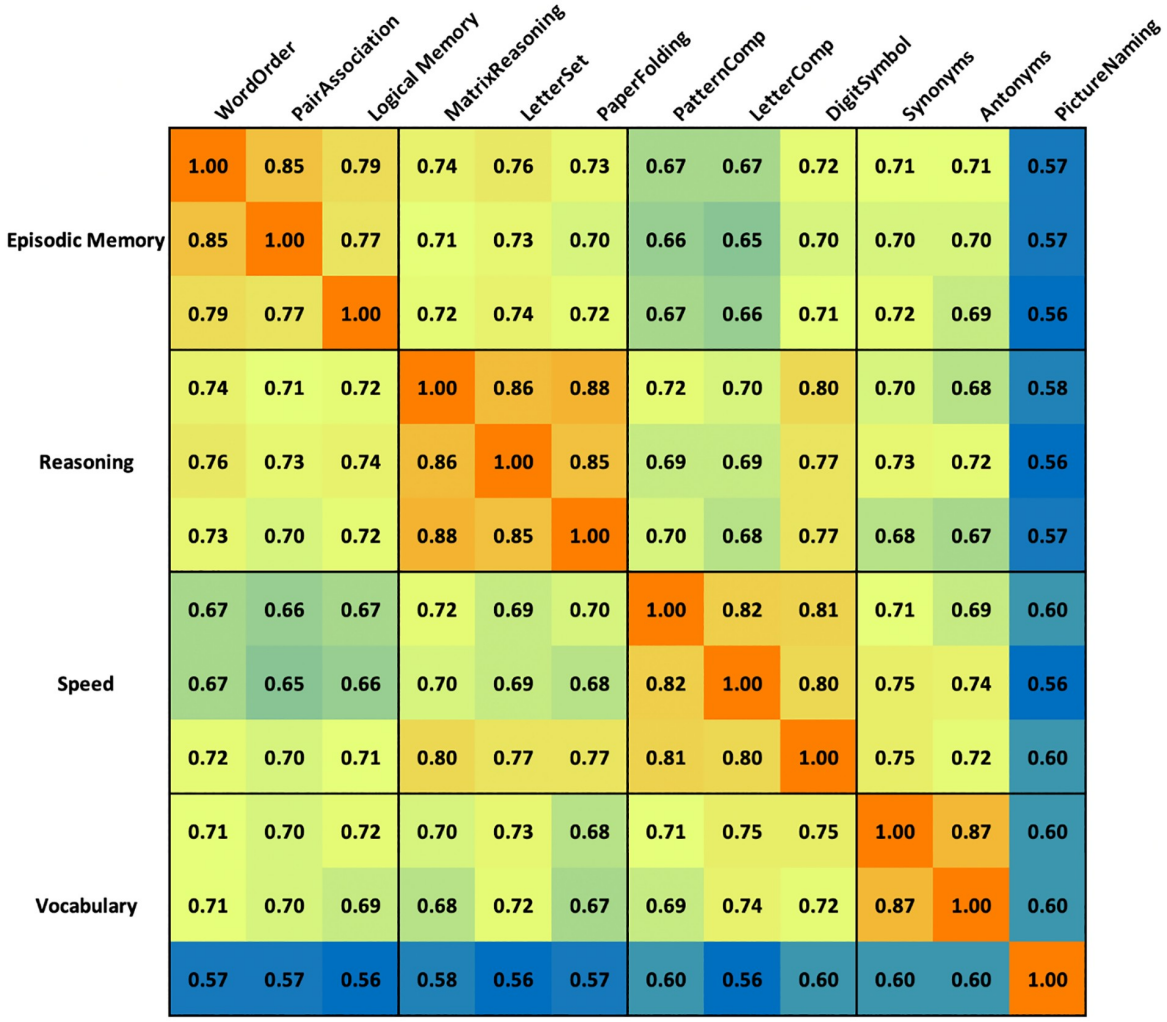
Color-coded correlation matrix of group level z-statistics. Warm colors indicate larger positive correlations. Cool colors indicate smaller positive correlations.

As in the analogous behavioral data analysis, we compared the structure, loadings and statistical fit parameters CFI, TLI, SRMR, RMSEA, and the Chi-Square test for the three different models. As can be seen in Table 2 (bottom), while all 3 models demonstrated good fit, the 4- and 5-factor models had better CFI and TLI than the 3-factor model, and the 4-factor model was on par with the 5-factor model across the other fit statistics but is a more parsimonious model. The full correlation matrix of the group level z-statistics for 12 tasks is shown in Fig 2.

**Fig 3:**
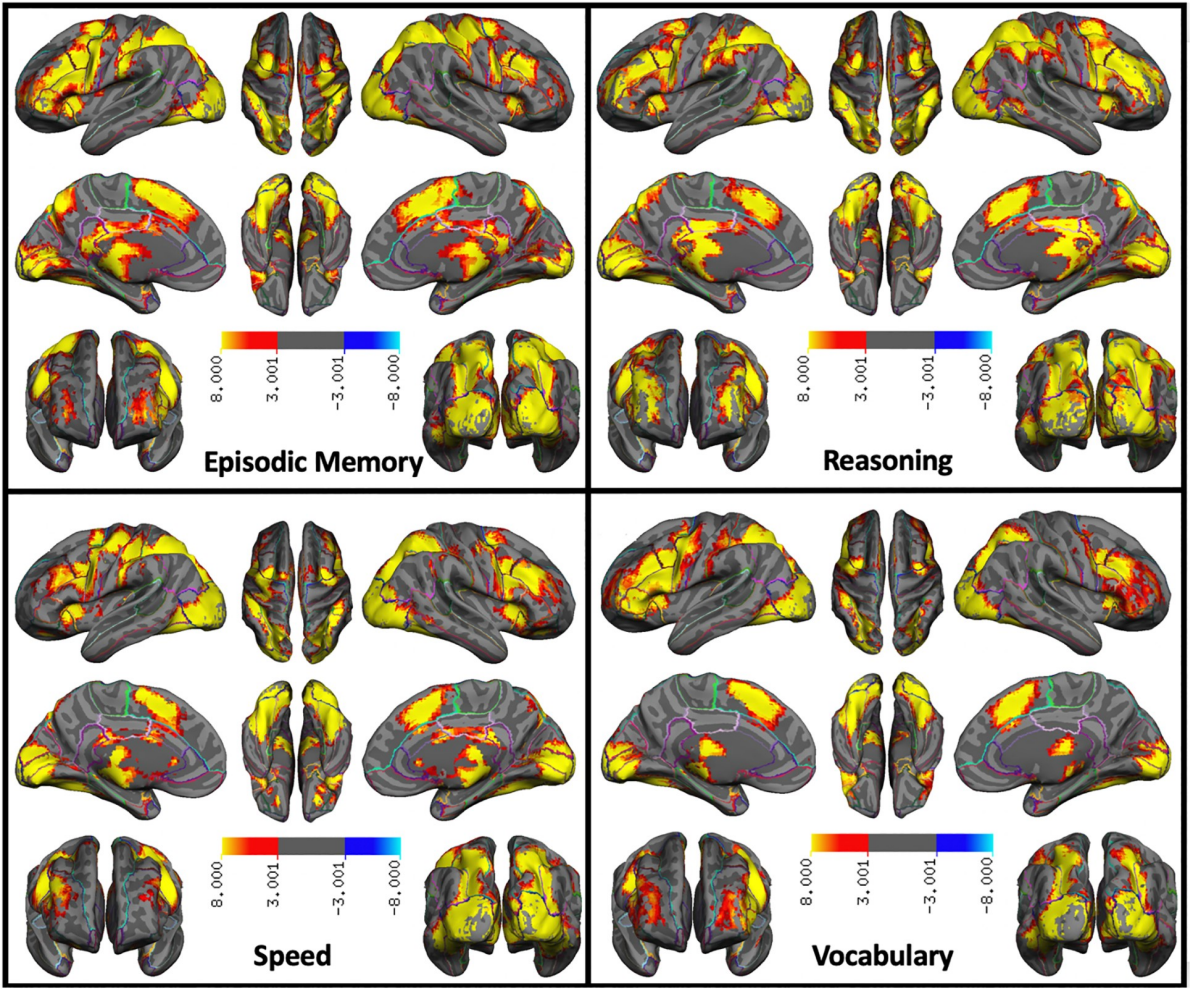
Patterns of BOLD activation showing significant topographic overlap within each cognitive domain.

The patterns of activation within each cognitive domain are shown in Fig 3. Only the Vocabulary pattern showed three distinct clusters ranging in size from 16,7000 voxels to 148 voxels; each other domain showed only one super-threshold cluster containing, in each case, more than 20,000 voxels. To better distinguish the brain regions within these large clusters, we used FSL’s autoaq tool to find percentages of the overlap in brain regions using Talairach Daemon Atlas. While the patterns all show strong activations in the cerebellum and posterior visual regions (BA17-19), they are more distinct in the frontal, parietal, and temporal lobes. The episodic memory retrieval pattern was dominated by bilateral activations in the middle frontal gyrus, the precuneus, the precentral gyrus, the inferior frontal gyrus, and the inferior parietal lobule. While the middle frontal gyrus also occupied the second largest proportion of the cluster in the reasoning pattern, larger overlaps were observed in the inferior parietal lobule and the superior frontal gyrus than for the other domains. For the speed pattern, with the cerebellum and the middle frontal gyrus also showing the top two largest overlaps, the third largest overlap was observed in the precentral gyrus. Vocabulary showed the most pronounced left lateralized prefrontal activation especially in the left middle and inferior frontal gyri. Cluster 2 in the vocabulary pattern resided in the right prefrontal areas and cluster 3 was in the left superior and middle temporal gyri. Table 3 lists the 10 brain regions that overlapped the most with significantly activated voxels from the group level clusters. S1–S5 Figs show.

**Table 3:** Top 10 brain regions with the largest overlap in each domain’s pattern from FSL’s autoaq using the Talairach Daemon Atlas. All Brodmann Areas shown are bilateral except where indicated by L/R.

Domain	# voxels	% overlap	Brain region	Brodmann Areas
**Episodic**	2167	8.72	Cerebellum	--
**Memory Retrieval**	1669	6.72	Middle Frontal Gyrus	6, 8, 9, 10, 46
	1501	6.04	Precuneus	7, 18L, 19, 31, 39L
total voxels	1232	4.96	Precentral Gyrus	4, 44
24,853	978	3.94	Lingual Gyrus	17, 18, 19
	966	3.89	Cuneus	7, 17, 18, 19, 23, 30
max Z = 19.0	856	3.46	Inferior Parietal Lobule	7, 39, 40
	838	3.37	Middle Occipital Gyrus	18, 19, 37L
Max X, Y, Z	786	3.16	Cingulate Gyrus	23, 24, 31, 32
[0–57–36]	736	2.96	Inferior Frontal Gyrus	6R, 9, 10L, 13, 44, 45, 46L, 47
**Reasoning**	2504	9.52	Cerebellum	--
	1898	7.21	Middle Frontal Gyrus	6, 8, 9, 10, 46
total voxels	1447	5.50	Precuneus	7, 18L, 19, 31, 39R
26,310	1365	5.19	Cuneus	7, 17, 18, 19, 23, 30
	1281	4.87	Inferior Parietal Lobule	7, 39, 40
max Z = 18.9	1147	4.36	Lingual Gyrus	17, 18, 19
	932	3.54	Middle Occipital Gyrus	18, 19, 37
Max X, Y, Z	879	3.34	Precentral Gyrus	4, 6, 9, 44
[–30–60–30]	809	3.07	Superior Frontal Gyrus	6, 8, 9, 10
	749	2.85	Cingulate Gyrus	23, 24, 31, 32
	585	2.22	Thalamus	--
**Speed**	2437	1.12	Cerebellum	--
	1423	5.74	Middle Frontal Gyrus	6, 8, 9, 10, 11, 46, 47
total voxels	1160	4,67	Precentral Gyrus	4, 6, 9, 44
24,806	1145	4.62	Cuneus	7, 17, 18, 19, 23, 30
	1083	4.37	Lingual Gyrus	17, 18, 19
max Z = 20.7	984	3.97	Precuneus	7, 18L, 19, 31
	966	3.89	Middle Occipital Gyrus	18, 19, 37
Max X, Y, Z	830	3.35	Inferior Parietal Lobule	7, 39R, 40
[21–90–3]	699	2.82	Inferior Frontal Gyrus	6R, 9, 11L, 13, 44, 45, 46R, 47
	601	2.42	Cingulate Gyrus	23, 24, 31R, 32
**Vocabulary**	2325	13.9	Cerebellum	--
Cluster 1	877	5.25	Left Middle Frontal Gyrus	6L, 8L, 9L, 10L, 11L, 46L, 47L
total voxels	852	5.1	Lingual Gyrus	17, 18, 19
16,700	779	4.66	Middle Occipital Gyrus	18, 19, 37L
	758	4.54	Cuneus	7L, 17, 18, 19, 23, 30
max Z = 20	690	4.13	Left Precentral Gyrus	4L, 6L, 9L, 44L
	653	3.91	Left Inferior Frontal Gyrus	9L, 10L, 11L, 13L, 44L, 45L, 46L, 47L
Max X, Y, Z	615	3.68	Precuneus	7, 18L, 19, 31
[24–87–6]	522	3.13	Fusiform Gyrus	18, 19, 20, 36L, 37
	494	2.96	Superior Frontal Gyrus	6, 8, 9L, 10L
Cluster 2	647	27.3	Right Middle Frontal Gyrus	6R, 8R, 9R, 19R, 11R, 46R, 47R
total voxels 2368	479	20.22	Right Inferior Frontal Gyrus	6R, 9R, 13R, 44R, 45R, 46R, 47R
max Z = 14.1	325	13.7	Right Precentral Gyrus	4R, 6R, 9R, 44R
Max X, Y, Z	164	6.9	Right Superior Frontal Gyrus	9R, 10R
[33 24 3]	114	4.8	Right Insula	13R, 47R
Cluster 3	76	51.4	Left Middle Temporal Gyrus	21L, 22L
total voxels 148	44	29.7	Left Superior Temporal Gyrus	22L
max Z 7.4				
Max X, Y, Z				
[–48–42 6]				

Voxels that are significantly more active during one domain as compared to the other 3 domains, for all four domains, as well voxels that were significantly active across all 4 domains.

### Brain-behavior relationship

**Fig 4:**
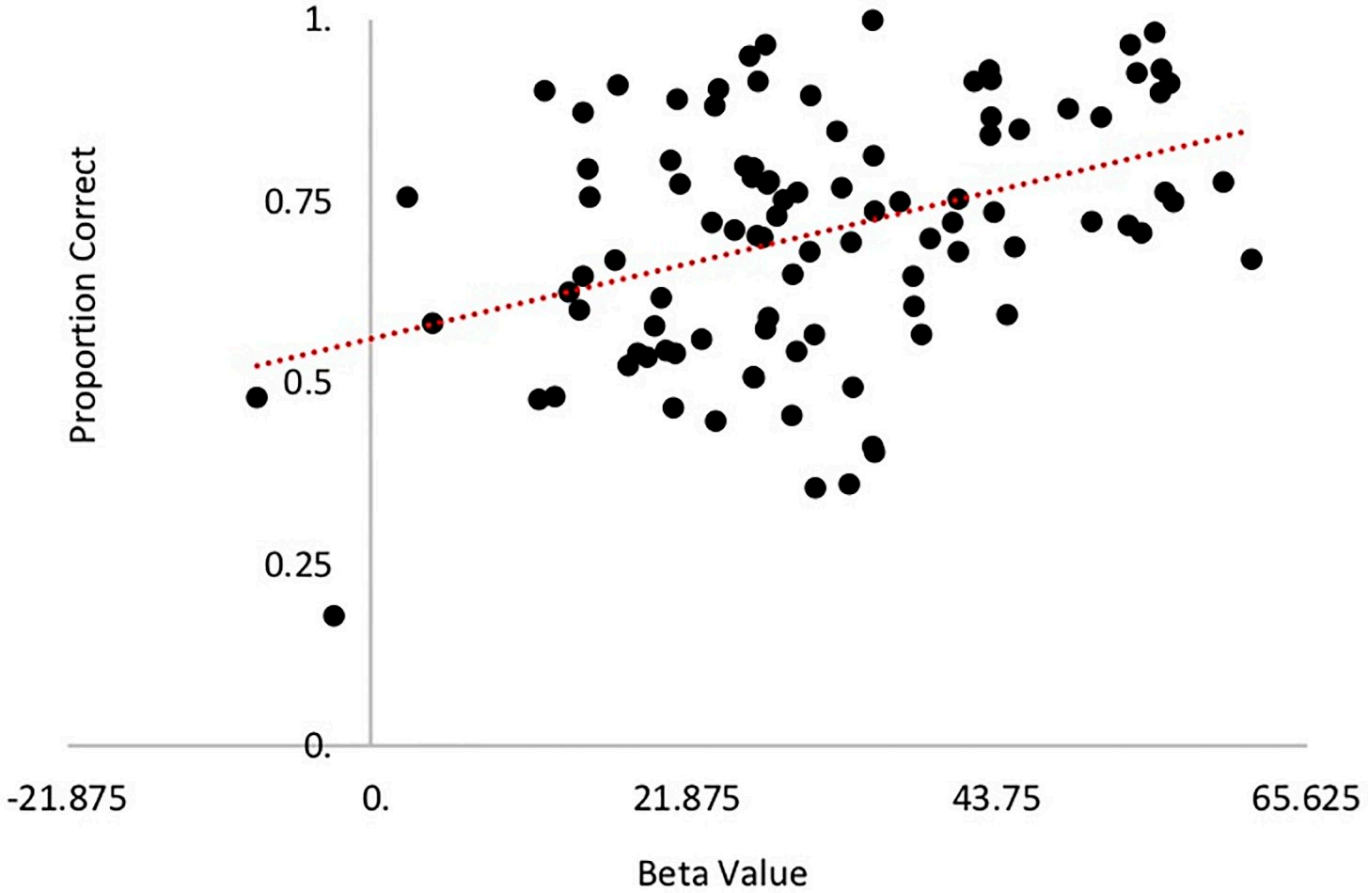
Relationship between average beta-value and behavioral performance across the three episodic memory tasks.

As the imaging data did not regress performance in the GLM, any relationship between brain activity within each cognitive domain and average behavioral performance in the corresponding cognitive tasks was not predetermined. To explore whether the magnitude of brain activity within each cognitive domain was associated with behavioral performance within that domain, the beta values across the three tasks found to constitute each cognitive domain were extracted from the group level mask for each participant, and these parameter estimates were correlated with each participant’s averaged domain-level behavioral performance. We found a significant association between brain activity and behavior in the episodic memory retrieval domain (r = .310, *p* = .002), such that participants who activated the memory network to a greater extent also performed better (had higher accuracy). The removal of one outlier who exhibited an average beta value that was almost 5 standard deviations above the mean value strengthened, rather than mitigated, this relationship (r = .396, *p* < .001), which is shown in Fig 4. For the other domains, no such relationship between brain activity and performance was found (reasoning (r = .055, *p* = 585); speed of processing (r = .04, *p* = .219); vocabulary (r = .118, *p* = .210). This result mirrors that found by [12], in which Principal Component Analysis was used to derive spatial covariance patterns of brain activity in the same sample. There, while all four cognitive domains were “forced” to find an optimal pattern that predicted behavior, the memory pattern exhibited both the highest out of sample replication probability with 100% significant correlations, which “speaks to the success of achieving a unifying neural account of both domain specificity and behavioral performance” (p. 13).

## Discussion

In the current study, we took a bottom-up approach to uncover the latent behavioral and brain-based networks of particular behavioral cognitive operations. To this end, we tested a large sample of younger adults on 12 cognitive tasks while they underwent fMRI scanning. We first used EFA to determine the best latent structure of the behavioral performance. We then applied a similar approach to extract the best-fitting latent pattern of neural activations, based on pairwise correlations of z-statistics in overlapping topographic regions derived from a GLM analysis of each task’s activations. The resulting correlation matrix was subjected to a structural equation model in which 3, 4, and 5 factor models were tested for fit.

While all 3 models fit the behavioral data well, the 4- and 5-factor models were better for the imaging data. In the 3-factor behavioral model, the memory retrieval and reasoning indicators combined to produce one factor, whereas in the imaging data, it was the speed and vocabulary indicators that combined, while the memory and reasoning indicators remained distinct. Likewise, for the 5-factor model, the imaging data dissociated picture naming from the other two tasks related to vocabulary. This is not surprising, as the picture naming task was quite different methodologically from the other 11 tasks, as it required participants to verbally name pictures rather than indicating their response using to touch pad. Interestingly, although the 5-factor model showed a distinct brain network for this task, in the behavioral models this task did not separate from the other vocabulary tasks. Despite differences in the patterns of loadings for the 3- and 5- factor models for the behavioral and imaging data, the 4-factor model showed remarkable consistency across the two modalities, where each set of data revealed an episodic memory retrieval, a reasoning, a vocabulary and a speed latent variable with reasonable fit-indices. Finally, to explore the relationships between the behavioral and neuroimaging latent structures, performance within each factor was correlated with the average beta value from each factor’s pattern of topographic overlap. Interestingly, only episodic memory performance correlated with strength of activation: those individuals who recruited this network to a greater extent also showed better performance on the tasks.

While the decision to adopt one solution over the others is somewhat arbitrary when more than one model produces adequate fit statistics, we chose to focus on the 4-factor solution because of the consistency of the neural and behavioral data loadings, it’s parsimony, and finally because aligns well with previous work that has attempted to explore cognitive structures utilizing different analytic techniques (see [16,35]). Each of the latent topographic neural networks that resulted from the four-factor model exhibited large and distributed clusters of activation. Some areas of activation, including those in the cerebellum, an area recognized to be involved in linguistic processing [36] and mental calculations [37], and posterior visual regions (BA17-19), appeared to be non-specific to cognitive function, as they were present across all 4 cognitive domain (see S5 Fig). This domain-general activation may be due to commonalities across all 12 of the tasks, including the use of visually presented stimuli. On the other hand, there were a number of regions that appeared to be domain specific. The episodic memory retrieval covariance pattern consisted of prefrontal (BA 10, 45, 47) and parietal regions (BA 39 and 40), as well as the precuneus, an area that has been shown in non-human primate studies to be highly connected to both cortical and subcortical structures [38]. All of these areas have consistently been associated with episodic memory retrieval [39–43]. Since the memory tasks relied on word recall and reading paragraphs, activation in semantic processing areas were also observed, such as the left inferior frontal gyrus (BA 44 and 45) as was reported for a word recall task [44]. The reasoning pattern showed peak activations in the inferior parietal lobule (BA 39 and 40), an area shown in early lesion studies to be associated with the ability to perform mental calculations [45,46] and in a more recent study on parietal focal lesions [47]. Activation in this area has also been associated with the attentional processes that enable orientation in 3-dimensional space [48]. While the other three domain patterns showed bilateral prefrontal activations, the vocabulary covariance pattern showed pronounced left lateralized prefrontal activations involving BA 8 of the prefrontal cortex, an area associated with numerous language-related abilities including speech motor programming [49], language processing [50] and translation [51] and sentence generation [49], as well as Broca’s Area (BA 44 and 45) in the inferior frontal gyrus, highly implicated in language (see [52] for a recent review). Using four different tasks that all required semantic processing, [53] showed activations common to all four tasks in the left inferior frontal and the left middle temporal gyri, consistent with clusters 1 and 3 in the vocabulary covariance pattern reported here. Additional peaks were located in the insula (BA 13), associated in previous imaging studies with phonological processing [54] as well as speech tasks [55,56]. Finally, the speed of processing network exhibited distributed activation in all lobes of the brain, with cluster peaks in primary and secondary visual cortex (BA 17 and 18). A study by Marcar and colleagues [57] reported increased BA 18 activity for simple versus complex shape discrimination, suggesting that this area may be sensitive to features in stimuli such as edges and vertices. A study by Fokin and colleagues [58] also showed that these areas were involved in processing of incomplete, but ordered (as opposed to chaotic) patterns. The precentral gyrus showed the third highest percentage of overlap in the speed pattern, consistent with its essential role in motor processes [59].

## Conclusion

Our goal was to first determine whether specific cognitive functions can be isolated using an unconstrained, bottom up data analysis approach, and then to test whether these latent cognitive domains of cognition measured behaviorally also map to distinct topographic patterns of neural activity. To this end, a large sample of participants underwent fMRI while they completed a set 12 cognitive tasks that were based upon paper and pencil tests previously used in studies of several thousand adults across the adult lifespan who were administered extensive batteries of cognitive tasks [60–62]. In administering this set of tasks, we attempted to avoid the possibly idiosyncratic features of any individual tasks, and instead identify broad and replicable cognitive aspects common to several tasks. The results of this study show that, indeed, different cognition tasks, thought to tap broader ontologies of cognitive function, can be specified using analytic techniques that are data-drive and free of construct assumptions, both behaviorally and neurally.

## Supporting information

S1 Data (TXT)

S2 Data (TXT)

S1 Fig (PNG)

S2 Fig (PNG)

S3 Fig (PNG)

S4 Fig (PNG)

S5 Fig (PNG)
