# Supplementary figures and images for: Towards an ontology of cognitive processes and their neural substrates: A structural equation modeling approach
Source: PLoS One. 2020 Feb 10;15(2):e0228167. doi: 10.1371/journal.pone.0228167 (PMC7010254; doi:10.1371/journal.pone.0228167)

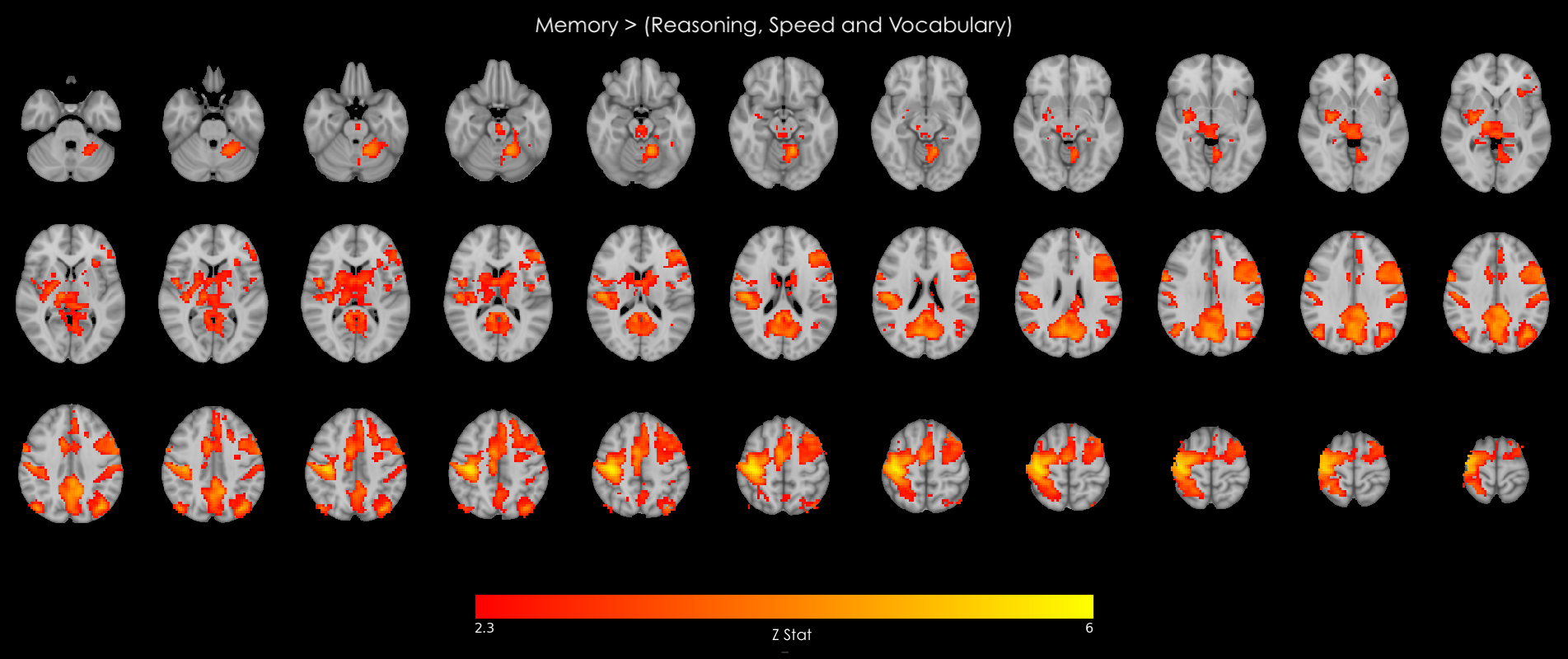

Supplement: S1 Fig — (PNG) [file pone.0228167.s003.png]

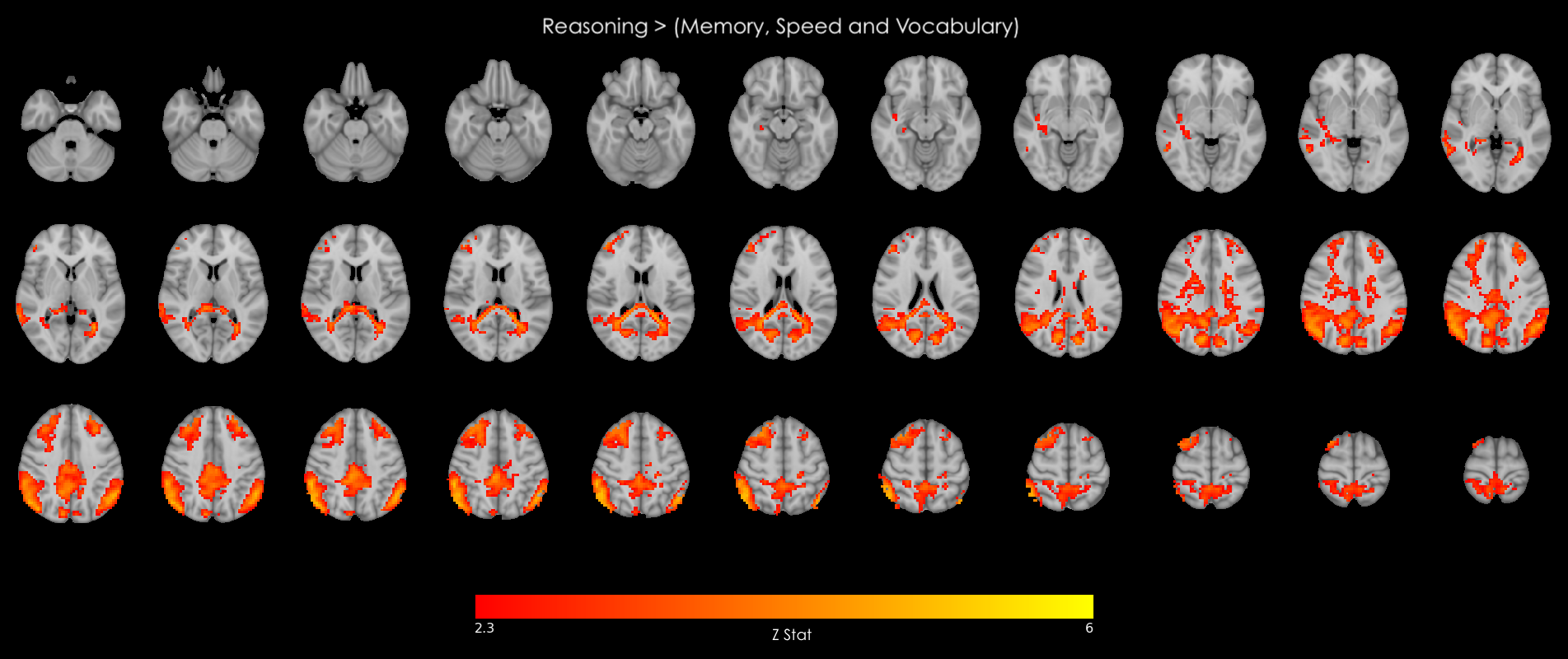

Supplement: S2 Fig — (PNG) [file pone.0228167.s004.png]

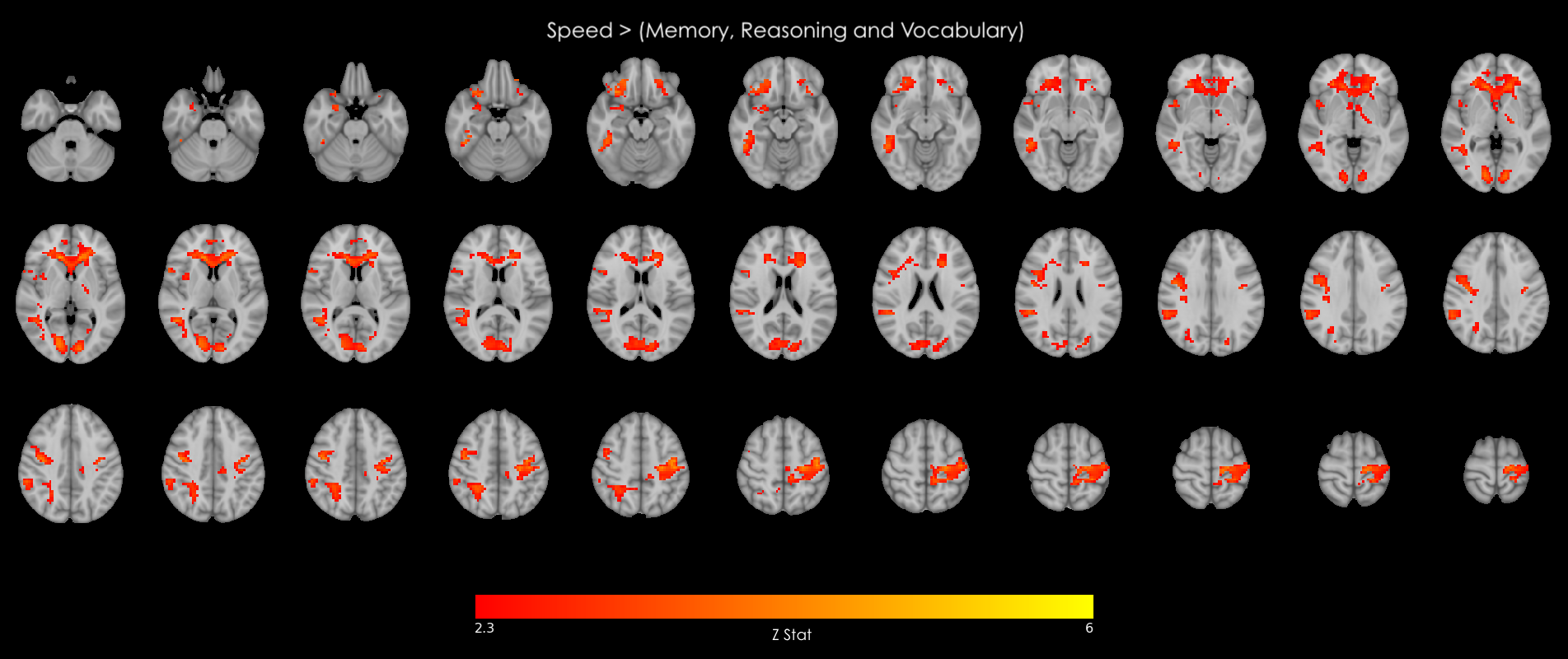

Supplement: S3 Fig — (PNG) [file pone.0228167.s005.png]

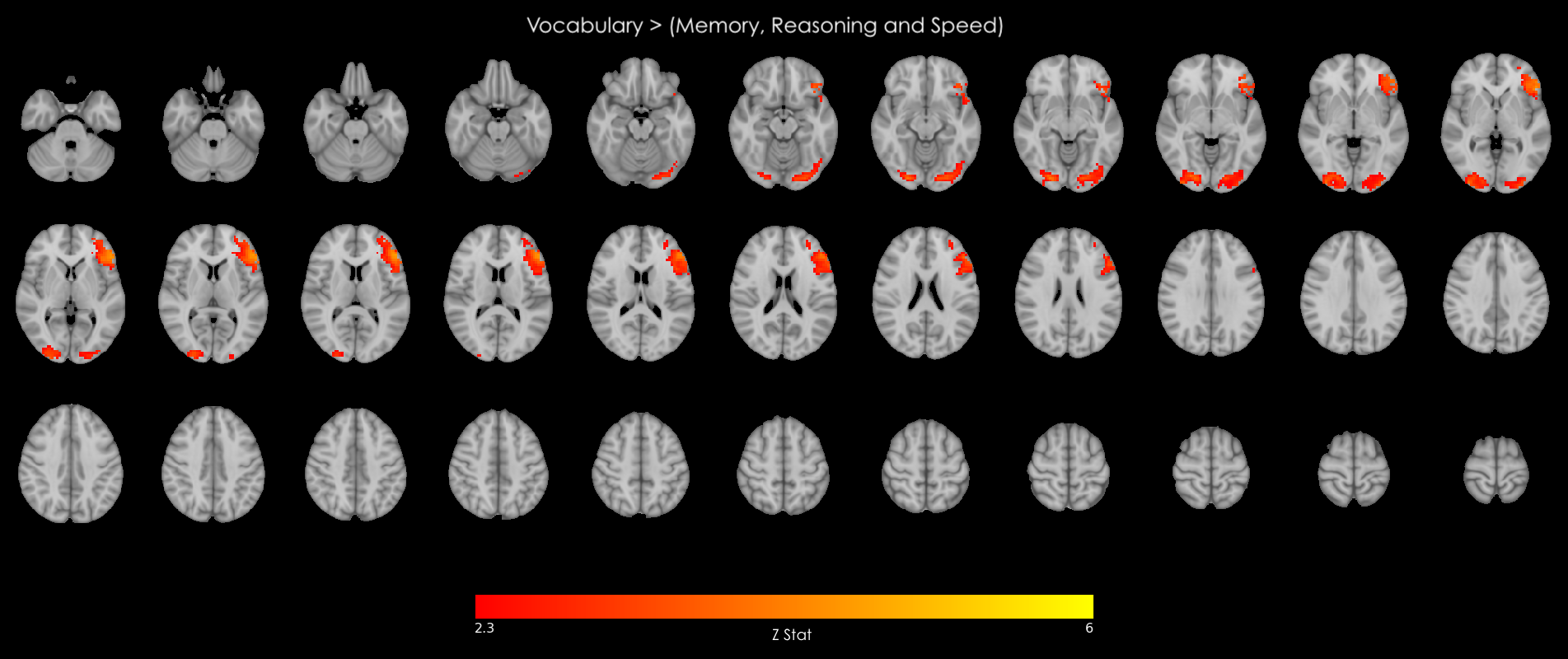

Supplement: S4 Fig — (PNG) [file pone.0228167.s006.png]

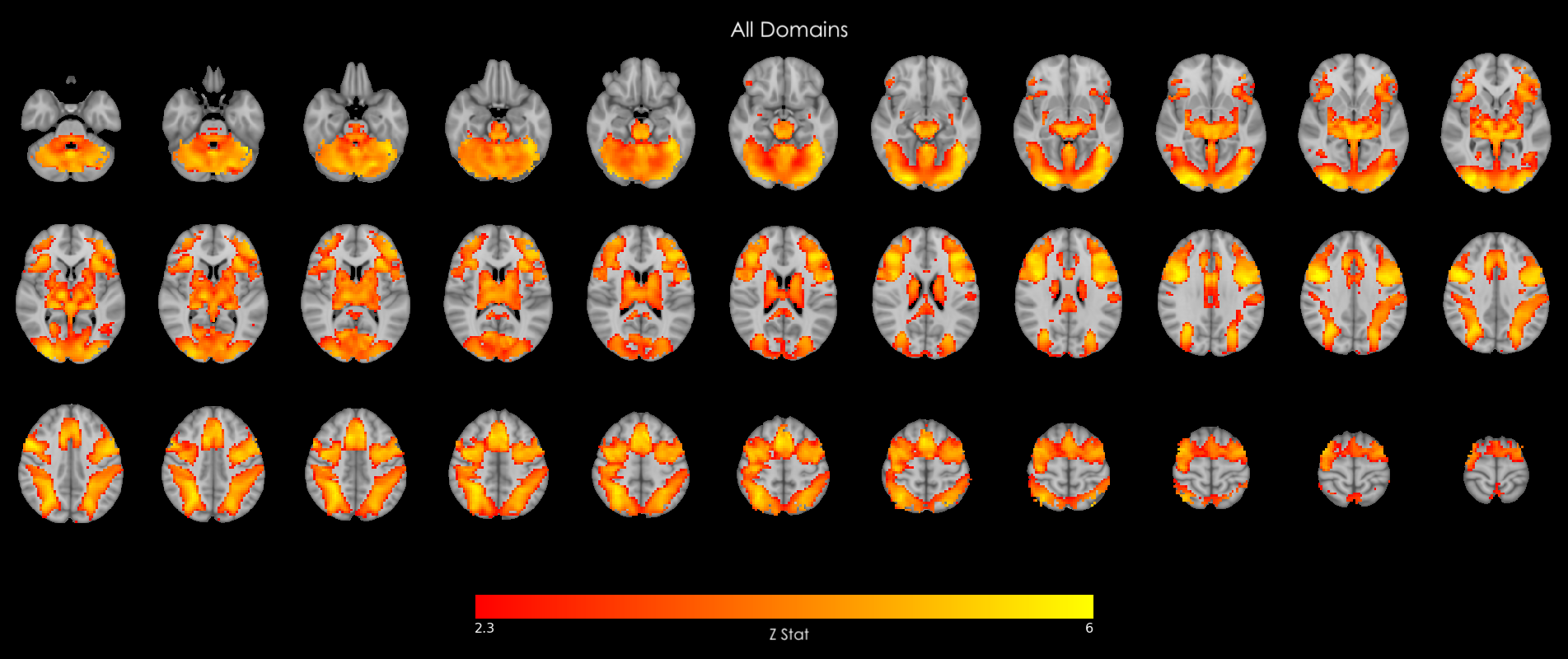

Supplement: S5 Fig — (PNG) [file pone.0228167.s007.png]
